# Human PD-1^hi^CD8^+^ T Cells Are a Cellular Source of IL-21 in Rheumatoid Arthritis

**DOI:** 10.3389/fimmu.2021.654623

**Published:** 2021-03-19

**Authors:** Kazuhiko Higashioka, Motoki Yoshimura, Takahide Sakuragi, Masahiro Ayano, Yasutaka Kimoto, Hiroki Mitoma, Nobuyuki Ono, Yojiro Arinobu, Makoto Kikukawa, Hisakata Yamada, Takahiko Horiuchi, Koichi Akashi, Hiroaki Niiro

**Affiliations:** ^1^ Department of Medicine and Biosystemic Science, Graduate School of Medical Sciences, Kyushu University, Fukuoka, Japan; ^2^ Department of Orthopaedic Surgery, Graduate School of Medical Sciences, Kyushu University, Fukuoka, Japan; ^3^ Department of Internal Medicine, Kyushu University Beppu Hospital, Oita, Japan; ^4^ Department of Medical Education, Faculty of Medical Sciences, Kyushu University, Fukuoka, Japan; ^5^ Department of Arthritis and Immunology, Graduate School of Medical Sciences, Kyushu University, Fukuoka, Japan

**Keywords:** rheumatoid arthritis, IL-21, CD8, T cells, PD-1

## Abstract

**Background:**

Rheumatoid arthritis (RA) is a prototypical autoantibody-driven autoimmune disease in which T-B interactions play a critical role. Recent comprehensive analysis suggests that PD-1^+^CD8^+^ T cells as well as two distinct IL-21-producing PD-1^+^CD4^+^ T cell subsets, follicular helper T (Tfh) and peripheral helper T (Tph) cells, are involved in the pathogenesis of RA. Herein, we aimed to clarify a generation mechanism of IL-21-producing CD8^+^ T cells in humans, and to characterize this novel subset in patients with RA.

**Methods:**

CD8^+^ T cells in the peripheral blood (PB) and synovial fluid (SF) of healthy control (HC) and patients with RA were subject to the analysis of IL-21 mRNA and protein. We evaluated the surface marker, cytokine and transcription profiles of IL-21-producing CD8^+^ T cells in HCPB, RAPB and RASF.

**Results:**

IL-21-producing CD8^+^ T cells were enriched in the CD45RA^-^(memory) PD-1^+^, especially PD-1^hi^ subpopulation, and IL-12 and IL-21 synergistically induced IL-21 production by naïve CD8^+^ T cells. Memory PD-1^hi^CD8^+^ T cells in HCPB facilitated plasmablast differentiation and IgG production in an IL-21-dependent manner. In addition, PD-1^hi^CD8^+^ T cells in RASF and RAPB produced large amounts of IL-21 and were characterized by high levels of CD28, ICOS, CD69, HLA-DR, and CCR2 but not CXCR5. Furthermore, PD-1^hi^CD8^+^ T cells expressed high levels of transcripts of *MAF* and *PRDM1*, a feature observed in Tph cells.

**Conclusions:**

Identification of IL-21-producing PD-1^hi^CD8^+^ T cells expands our knowledge of T cell subsets with B helper functions in RA. Selective targeting of these subsets could pave an avenue for the development of novel treatment strategies for this disease.

## Introduction

Rheumatoid arthritis (RA) is a prototypical autoimmune disease characterized by joint inflammation and bone destruction (1). The emergence of autoantibodies (auto-Abs) such as anti-citrullinated protein antibodies (ACPA) and rheumatoid factors (RF) in the preclinical stage of RA underscores the autoimmune-driven process of this disease (2). In the clinical stage, these auto-Abs can form immune complexes in the joints in which attraction and activation of various immune cells take place, culminating in synovial inflammation and bone destruction (3).

Mounting evidence including genome-wide association studies suggests that RA is an MHC class II-associated disease in which CD4^+^ T cells play a critical role (4). Follicular helper T (Tfh) cells are a CXCR5^+^PD-1^+^CD4^+^ T cell subset with abundant production of IL-21 that in turn helps the generation of memory B cells and plasma cells in the germinal center (GC) (5). Of note, humans have Tfh cells in the peripheral blood (PB) termed circulating Tfh (cTfh) (6). cTfh cells harbor the potential to support antibody secretion (7) and altered numbers of cTfh cells have been reported in patients with autoimmune diseases including RA (8–12).

Previous studies, however, showed that CXCR5^-^PD-1^+^CD4^+^ T cells can also produce IL-21 that is instrumental in the generation of ectopic lymphoid structures in RA synovium (13, 14). Thus, these findings pose the question of which CD4^+^ T cell subset (CXCR5^+^ versus CXCR5^-^) is critical in the pathogenesis of RA. To address this issue, Rao et al. recently characterized the features of Tfh and CXCR5^-^PD-1^+^CD4^+^ T cells, and coined the latter ‘peripheral helper T’ (Tph) cells (15, 16). Despite possession of their B cell helper functions *via* IL-21 production, Tfh and Tph cells exhibit distinct features. Tph cells lack CXCR5, but instead express high levels of CCR2 that allows the recruitment of this subset to inflammatory sites. In addition, two subsets exhibit both similar and different expression patterns of transcription factors (TFs). Both cells highly express MAF (15, 17), while Tph cells highly express BLIMP1, a transcription factor typically downregulated in Tfh cells (15, 17).

Since RA is an MHC II-associated disease, the role of CD8^+^ T cells in this disease has attracted relatively little attention. However, CD8^+^ T cells comprise ~40% of all T cells in RA synovium and the abundance of these cells in SF and PB is closely associated with disease activity in RA (18, 19). CD8^+^ T cells are generally considered a prototypical cytotoxic cell type. A recent high-quality study using single-cell transcriptomics and mass-cytometry identified several distinct CD8^+^ T cell subsets in the synovium of patients with RA (20). Of note, CD8^+^ T cells constitute PD-1^+^ and PD-1^-^ subpopulations; the latter only enriches granzyme-producing cytotoxic cells. What then does the former (PD-1^+^CD8^+^ T cell) do in this case? Apart from their cytotoxicity, several lines of evidence suggest that CD8^+^ T cells can be another source of IL-21. In mice, IL-6 induces IL-21-producing CD8^+^ T cells that help in the production of virus-specific IgG Abs (21). In humans, IL-21-producing CD8^+^ T cells are detected in the tissues of patients with nasal polyps and Hodgkin lymphoma (22, 23). Interestingly, IL-21-producing CD8^+^ T cells in polyp tissues express PD-1 and ICOS at high levels and promote IgG production *in vitro* (22). Given that CD8^+^ T cells and B cells are abundant in lymph nodes of early RA patients (24) and CD8^+^ T cells play a role in modulating ectopic GC formation in RA (25, 26), PD-1^+^CD8^+^ T cells may play a pathogenic role in RA *via* IL-21 production. At present, however, it remains unknown how PD-1^+^CD8^+^ T cells are generated in humans and whether the features of these cells could be similar to or distinct from Tfh and Tph cells in human autoimmune diseases such as RA.

In this study, we demonstrate that IL-21-producing CD8^+^ T cells were enriched in the CD45RA^-^(memory) T cells, especially in the PD-1^hi^ subpopulation, whereas granzyme B-producing CD8^+^ T cells were abundant in the terminal effector subpopulation. IL-12 and IL-21 synergistically induced IL-21 production by naïve CD8^+^ T cells. Memory PD-1^hi^ CD8^+^ T cells in HCPB facilitated plasmablast differentiation and IgG production in an IL-21-dependent manner. In addition, PD-1^hi^CD8^+^ T cells in RASF and RAPB produced large amounts of IL-21 and were characterized by high levels of CD28, ICOS, CD69, HLA-DR, and CCR2 but not CXCR5. Furthermore, PD-1^hi^CD8^+^ T cells expressed high levels of transcripts of *MAF* and *PRDM1*, a feature observed in Tph cells. Together, these findings suggest that PD-1^hi^CD8^+^ T cells in RASF play, in concert with Tfh/Tph cells, a pivotal role in the pathogenesis of RA.

## Materials and Methods

### Patients and Controls

We studied 18 patients with RA who fulfilled the 1987 American College of Rheumatology classification criteria and ACR & EULAR 2010 classification criteria. Eighteen synovial fluid (SF) samples were obtained from the patients. Patient details are shown in **Supplementary Table 1**. Informed consent was obtained from all subjects in accordance with the Declaration of Helsinki. The Institutional Review Board of Kyushu University Hospital approved all research on human subjects (No 29-544). We obtained information from the medical records of the patients, including clinical manifestations, laboratory findings and medication history.

### Reagents

Dynabeads Human T-Activator CD3/28 was purchased from Invitrogen (Carlsbad, CA, USA). An affiniPure F (ab’)^2^ Fragment Goat Anti-Human IgA/IgG/IgM (H+L) (anti-BCR, 10 μg/ml) was purchased from Jackson ImmunoResearch (West Grove, PA, USA). Recombinant human cytokines (IL-12 (50 ng/ml), IFN-γ (20 ng/ml), IL-4 (20 ng/ml), IL-6 (50 ng/ml), IL-10 (10 ng/ml), IL-21 (50 ng/ml), TGF-β (50 ng/ml), IL-2 (100 U/ml), IL-15 (1 ng/ml) and recombinant human IL-6 Receptor (100 ng/ml) were obtained from R&D Systems (Minneapolis, MN, USA). CpG oligonucleotide type B (0.1 μM) was purchased from Gene Design Inc. (Osaka, Japan). Phorbol myristate acetate (PMA) and Ionomycin were purchased from Calbiochem (Nottingham, UK). A human monoclonal antibody (mAb) against IL-21 (anti-IL-21, used at the dose of 10 μg/ml) was purchased from Mabtech AB (Nacka Strand, Sweden).

### Isolation and Cell Sorting of T and B Cell Subsets

Peripheral blood mononuclear cells (PBMCs) were obtained using a density centrifugation with LSM (MP Biomedicals, LLC, Santa Ana, CA, USA). CD4^+^ T cells, CD8^+^ T cells and CD19^+^ B cells were isolated by positive selection with anti-CD4, CD8 and CD19 microbeads and a MACS magnetic cell sorting system (Miltenyi Biotec, Bergisch Gladbach, Germany). Isolated CD4^+^ T cells, CD8^+^ T cells and CD19^+^ B cells exhibited greater than 99.5% viability and more than 95% purity, confirmed by flow cytometry. Cells were stained with mouse or rabbit mAbs against human CD3, CD4, CD8, CD19, CD20, CD27, CD28, CD45RA, CD69, CD95, CD192 (CCR2), CD197 (CCR7), ICOS (CD278), PD-1 (CD279) and HLA-DR (all from BioLegend, San Diego, CA, USA). Zombie Dyes purchased from BioLegend were used to exclude dead/dying cells in intracellular cytokine staining. Naïve CD8^+^ T cells were defined as CD45RA^+^CCR7^+^, memory CD4^+^ T cells as CD45RA^-^, memory CD8^+^ T cells as CD45RA^-^, and memory CD19^+^ B cells as CD27^+^. For some experiments, CD45RA^+^ CD8^+^ T cells, CD45RA^-^PD-1^-^CD8^+^ T cells, CD45RA^-^PD-1^int^CD8^+^ T cells, CD45RA^-^PD-1^hi^CD8^+^ T cells and CD45RA^-^PD-1^hi^CD4^+^ T cells were purified by flow cytometry.

### Quantitative Real-Time Polymerase Chain Reaction

Total RNA was extracted from primary CD8^+^ T cells using Isogen II reagent (Nippon Gene, Tokyo, Japan). Quantitative real-time PCR was performed in the MX3000P Sequence Detector (Agilent technologies, Santa Clara, CA, USA). TaqMan target mixes for *IL-21* (Hs00222327_m1), *MAF* (Hs04185012_s1), *BCL6* (Hs00153368_m1), *PRDM1* (Hs00153357_m1), *TCF7* (Hs01556515_m1) were all purchased from Applied Biosystems. 18S ribosomal RNA was separately amplified in the same plate as an internal control for variation in the amount of cDNA in PCR. The collected data were analyzed using Sequence Detector software (MX3000P). Data were expressed as the fold change in gene expression relative to the expression in control cells.

### Intracellular Staining of IL-21, IFN-γ and IL-17

Phorbol 12-myristate 13-acetate (PMA, 50 ng/ml, Calbiochem, Nottingham, UK), ionomycin (1 μM, Calbiochem) and Golgi Stop (Brefeldin-A, eBioscience, Carlsbad, CA, USA) were added 6 h before staining. Cell surface staining was performed before intracellular cytokine staining for 20 min. After washing two times, fixation/permeabilization buffer (BD Biosciences) was added to fix the cells for 20 min. Antibodies to detect IL-21, IFN-γ and IL-17 (Biolegend) were added to cell suspension and intracellular staining was performed for 15 min. After washing two times, cells were analyzed by FACS Aria II (BD Biosciences). We evaluated IL-21 production in CD8^+^ T cells in IFN-γ ^+^, IFN-γ ^-^, IL-17^+^ and IL-17^-^ fractions to investigate the relationship between IL-21-producing cells and IFN-γ/IL-17-producing cells.

### Co-Culture Experiments

Upon stimulation of PBMCs with CD3/28 beads for 72 h, CD45RA^+^CD8^+^ T cells, CD45RA^-^PD-1^hi^ CD8^+^ T cells and CD45RA^-^PD-1^hi^ CD4^+^ T cells were purified by flow cytometry. Memory B cells were co-cultured with purified CD45RA^+^CD8^+^ T cells, CD45RA^-^PD-1^hi^CD8^+^ T cells and CD45RA^-^PD-1^hi^CD4^+^ T cells for 6 days with anti-BCR (10 μg/ml), CD3/28 beads and CpG (0.1 μM). CD19^+^ B cells and T cells at a ratio of 2:1 (3.2×10^5^ cells/mL: 1.6×10^5^ cells/mL) were cultured in a 96-well plate and the expression of surface markers including CD27 and CD38 in CD19^+^ cells was analyzed by flow cytometry.

### Enzyme-Linked Immunosorbent Assay (ELISA)

Pre-stimulated T cell and memory B cells were co-cultured under the conditions mentioned above and IgG production in the culture supernatants was measured using an IgG (Total) Human ELISA kit (Invitrogen, Carlsbad, CA, USA), according to the manufacturer’s instructions.

### Statistical Analysis

Numerical data in the *in vitro* experiments were presented as mean of the different experiments and standard error of the mean (SEM). Multiple group comparisons were analyzed using the Kruskal-Wallis test. The significance of the differences was determined by Student’s t-test for comparing differences between two groups. Numerical data in patient-sample analyses were presented as mean, and the significance of differences (SD) was determined by Student’s t-test or nonparametric Mann-Whitney U-test according to distributions. The correlations between two groups were analyzed using Spearman’s correlation coefficient. For all tests, *P* values less than 0.05 were considered significant. All analyses were performed using GraphPad Prism 8 (Prism, La Jolla, CA USA).

## Results

### CD8^+^ T Cells Committed to Produce IL-21 Exist in PB

**Figure 1:**
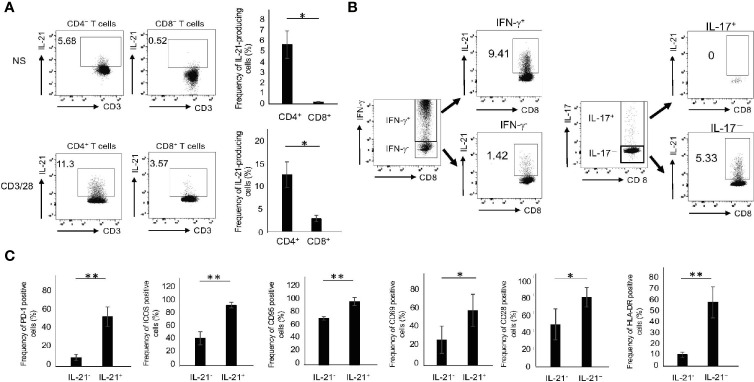
The phenotype of IL-21-producing CD8^+^ T cells. **(A)** PBMCs from HCs were stimulated in the absence or presence of CD3/28 beads for 72 h and then IL-21 production from CD4^+^ and CD8^+^ T cells was analyzed by intracellular staining (added PMA and Ionomycin for the last 6 h). Left panels are representative data on IL-21 production from CD4^+^ and CD8^+^ T cells stimulated with CD3/28 and the right graph summarizes the results (N=4). **(B)** PBMCs from HCs were stimulated under the same condition as **(A)**, and IFN-γ and IL-17 production from IL-21^+^ and IL-21^-^ CD8^+^ T cells was analyzed by intracellular staining. **(C)** PBMCs from HCs were stimulated under the same condition as **(A)**, and expression levels of PD-1, ICOS, CD95, CD69, CD28 and HLA-DR in IL-21^+^ and IL-21^-^ CD8^+^ T cells were analyzed by flow cytometry. The graph summarizes the results (N=4). *P < 0.05, **P < 0.01, NS, no stimulation.

We first compared IL-21 production in CD4^+^ and CD8^+^ T cells from the peripheral blood of HCs (HCPB). The gating strategy for intracellular cytokine staining of T cells is shown in **Supplementary Figure 1A**. In the absence of stimuli, there were small numbers of CD4^+^ but few CD8^+^ T cells capable of producing IL-21 (**Figure 1A**). Upon CD3/28 stimulation, however, they produced significant amounts of IL-21, although the levels in CD8^+^ T cells were still lower than those in CD4^+^ T cells (**Figure 1A**). The FMO control of IL-21 in CD4^+^ and CD8^+^ T cells with or without CD3/28 stimulation is depicted in **Supplementary Figure 1B**. We found that 72 h was the optimal time point for IL-21 production and all of the experiments were carried out in this condition thereafter (data not shown). In this situation, CD4^+^ and CD8^+^ T cells revealed an opposite trend in the production of IL-17 and IFN-γ: the former cells produced high levels of IL-17, while the latter cells produced high levels of IFN-γ (**Supplementary Figure 2**). Notably, IL-21 was predominantly produced by IFN-γ^+^ and IL-17^-^ CD8^+^ populations (**Figure 1B**). We also determined several surface markers that are associated with activated Tfh/Tph cells (5, 6, 15, 16) in IL-21-producing CD8^+^ T cells in HCPB and found that the frequency of PD-1^+^, ICOS^+^. CD95^+^, CD69^+^, CD28^+^ and HLA-DR^+^ in IL-21^+^CD8^+^ T cells was significantly higher than that in IL-21^-^CD8^+^ T cells (**Figure 1C**). These results suggest that CD8^+^ T cells, like CD4^+^ T subsets, which are committed to produce IL-21, exist in human PB.

### IL-21-Producing CD8^+^ T cells Are Enriched in Memory PD-1^hi^ Fraction

**Figure 2:**
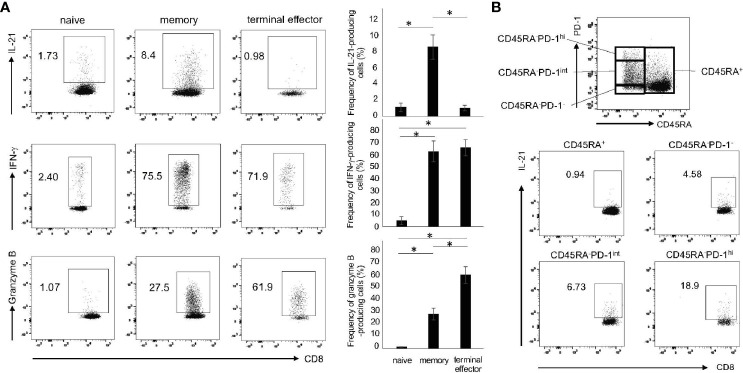
The subsets of IL-21-producing CD8^+^ T cells. **(A)** PBMCs from HCs were stimulated with CD3/28 beads for 72 h. Production of IL-21, IFN-γ and granzyme B in naïve (CD45RA^+^CCR7^+^), memory (CD45RA^-^) and terminal effector (CD45RA^+^CCR7^-^) cells was analyzed by intracellular staining (added PMA and Ionomycin for the last 6 h). Left panels are representative data and right graphs summarize the results (N=3). **(B)** PBMCs from HCs were stimulated under the same condition as **(A)**, and then analyzed by flow cytometry. The panel shows four fractions of CD8^+^ T cells (CD45RA^+^, CD45RA^-^PD-1^-/low^, CD45RA^-^PD-1^int^ and CD45RA^-^PD-1^hi^). The panels show IL-21 production from four populations including CD45RA^+^, CD45RA^-^PD-1^-/low^, CD45RA^-^PD-1^int^ and CD45RA^-^PD-1^hi^ CD8^+^ T cells by intracellular staining. *P < 0.05.

Given that CD8^+^ T cells comprise the subpopulations with distinct functions such as naïve, memory and effector cells (27), we determined the production of IL-21, IFN-γ and granzyme B in these subpopulations. As shown in **Figure 2A**, IL-21 was predominantly produced by memory (CD45RA^-^) CD8^+^ T cells. On the other hand, IFN-γ was produced by memory and terminal effector (CD45RA^+^CCR7^-^) CD8^+^ T cells, while granzyme B was produced by terminal effector CD8^+^ T cells (**Figure 2A**). Expression levels of PD-1 in Tfh/Tph cells correlate well with their potential for IL-21 production (28). We thus investigated IL-21 production in the four fractions: CD45RA^+^, CD45RA^-^PD-1^-^, CD45RA^-^PD-1^int^ and CD45RA^-^PD-1^hi^ (**Figure 2B**). Short-term treatment of PMA and ionomycin that are commonly used in intracellular staining did not affect PD-1 expression (data not shown). We found that 72 h was, again, the optimal time point for the induction of CD45RA^-^PD-1^hi^CD8^+^ T cells (data not shown). Interestingly, IL-21 production was largely confined to CD45RA^-^PD-1^hi^CD8^+^ T cells (**Figure 2B**). These results suggest that IL-21-producing CD8^+^ T cells are enriched in the memory, especially PD-1^hi^ fraction.

### IL-12 and IL-21 Are Critical in the Generation of IL-21-Producing CD8^+^ T Cells

**Figure 3:**
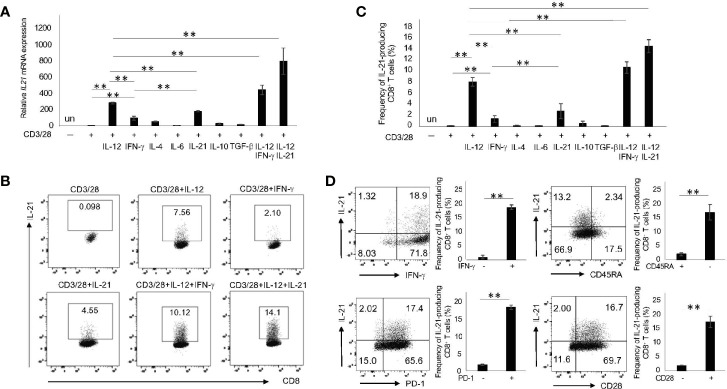
IL-21-producing CD8^+^ T cells are most efficiently induced by IL-12 and IL-21 in combination with CD3/28. **(A)** Naïve CD8^+^ T cells (CD45RA^+^CCR7^+^ CD8^+^ T cells) in HCs were purified by flow cytometry and then stimulated with the indicated cytokines in combination with CD3/28 beads for 72 h. Cells were harvested, and transcriptions of *IL21* were evaluated by qPCR. **(B)** Purified naïve CD8^+^ T cells in HCs were stimulated with the indicated cytokines for 5 days, and IL-21 production was analyzed by intracellular staining. Representative data are shown and summarized results are shown in **(C)** (N=4). **(D)** Naïve CD8^+^ T cells in HCs were stimulated with IL-12 and IL-21 in conjunction with CD3/28 beads. The panels show expression of CD45RA, PD-1, CD28 and IFN-γ on IL-21^+^ and IL-21^-^ CD8^+^ T cells. Representative data are depicted, and summarized graphs of the results are shown (N=4). **P < 0.01, un, undetected.

Given that naïve CD8^+^ T cells produced less IL-21 (**Figure 2A**), we hypothesized that a specific cytokine plays a pivotal role in their commitment towards IL-21 production. To this end, purified naïve CD8^+^ T cells were cultured with IL-12, IFN-γ, IL-4, IL-6, IL-21, IL-10 and TGF-β combined with CD3/28 stimulation and followed by the analysis of IL-21 production. As shown in **Figure 3A**, IL-12 and, to a lesser extent, IL-21 alone significantly up-regulated the levels of CD3/28-induced *IL21* mRNA in naïve CD8^+^ T cells. In addition, combination of IL-12 and IL-21 synergistically induced *IL21* mRNA expression in these cells. This trend observed in *IL21* mRNA was also true for IL-21 protein production (**Figures 3B, C** **)**. On the other hand, naïve CD8^+^ T cells produced significant IFN-γ upon CD3/28 stimulation only (**Supplementary Figure 3A, B** **)**. Interestingly, IL-12 enhanced, but IL-21 inhibited IFN-γ production. Combination of IL-12 and IL-21, however, induced IFN-γ production to a similar extent to IL-12 alone (**Supplementary Figure 3A, B** **)**. We thus reasoned that this combination induces the generation of IFN-γ^+^IL-21-producing CD8^+^ T cells and, indeed, this was the case (**Figure 3D**). Moreover, in this condition, IL-21-producing CD8^+^ T cells were mainly observed in the CD45RA^-^, CD28^+^ and PD-1^+^ fractions (**Figure 3D**). These results suggest that IL-12 and IL-21 are critical in the generation of IL-21-producing CD8^+^ T cells.

### Memory PD-1^hi^CD8^+^ T Cells Facilitate Plasmablast Differentiation and IgG Production in an IL-21-Dependent Manner

**Figure 4:**
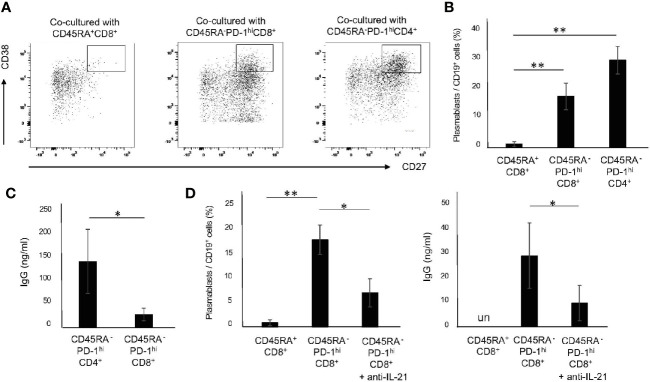
Memory PD-1^hi^CD8^+^ T cells promote the differentiation of B cells to plasmablasts and IgG production in an IL-21-dependent manner. **(A)** Upon stimulation of PBMCs from HCs with CD3/28 beads for 72 h, CD45RA^+^CD8^+^ T cells, CD45RA^-^ PD-1^hi^ CD8^+^ T cells and CD45RA^-^ PD-1^hi^ CD4^+^ T cells were purified by flow cytometry. Memory B (CD19^+^CD20^+^CD27^+^) cells were co-cultured with CD45RA^+^ CD8^+^ T cells, CD45RA^-^PD-1^hi^ CD8^+^ T cells and CD45RA^-^ PD-1^hi^CD4^+^ T cells in the presence of anti-BCR, CD3/28 beads and CpG for 6 days. Representative data on the expression of CD27 and CD38 are depicted in **(A)** and the results are summarized in **(B)** (N=4). **(C)** Comparison of IgG production from plasmablasts induced by co-cultured CD45RA^-^PD-1^hi^CD4^+^ T cells and CD45RA^-^PD-1^hi^CD8^+^ T cells. **(D)** Effect of IL-21 blockade on the frequency of plasmablasts and IgG production induced by co-cultured CD45RA^-^PD-1^hi^CD8^+^ T cells. *P < 0.05, **P < 0.01. un, undetected.

IL-21 is a critical cytokine for the differentiation of B cells to Ab-secreting cells (29, 30). Since 72 h was determined to be the optimal point for the induction of CD45RA^-^PD-1^hi^CD8^+^ T cells, we pre-stimulated PBMC with CD3/28 beads for 72 h. The CD45RA^+^CD8^+^ T cells, CD45RA^-^PD-1^hi^CD8^+^ T cells and CD45RA^-^PD-1^hi^CD4^+^ T cells were then purified by flow cytometry. Memory B cells were co-cultured with CD45RA^+^CD8^+^ T cells, CD45RA^-^PD-1^hi^CD8^+^ T cells and CD45RA^-^PD-1^hi^CD4^+^ T cells. Compared with CD45RA^+^CD8^+^ T cells, CD45RA^-^PD-1^hi^ CD8^+^ T cells as well as CD45RA^-^PD-1^hi^ CD4^+^ T cells induced large numbers of plasmablasts (CD19^+^CD27^+^CD38^hi^) (**Figures 4A, B** **)**. In addition, CD45RA^-^PD-1^hi^CD8^+^ T cells promoted IgG production, albeit to a lesser extent as compared with CD45RA^-^PD-1^hi^CD4^+^ T cells (**Figure 4C**). Furthermore, the anti-IL-21 antibody suppressed plasmablast differentiation and IgG production (**Figure 4D**). These results suggest that memory PD-1^hi^CD8^+^ T cells have the potential to facilitate the differentiation of memory B cells to plasmablasts and IgG production in an IL-21-dependent manner.

### Memory PD-1^hi^CD8^+^ T Cells Produce IL-21 in RAPB and RASF

**Figure 5:**
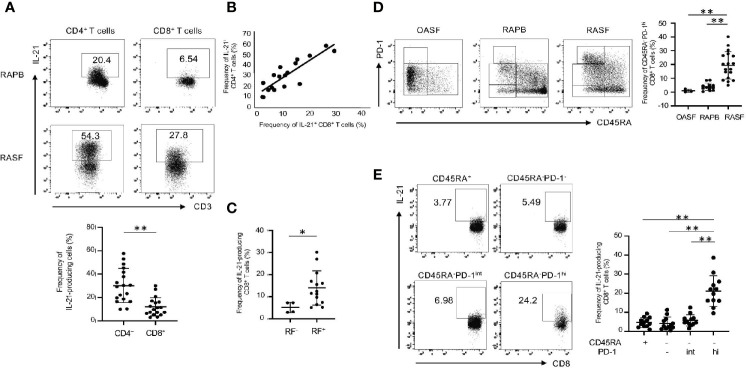
IL-21-producing CD8^+^ T cells are enriched in memory PD-1^hi^ fraction in RAPB and RASF. **(A)** The upper panels show the representative data regarding the percentage of IL-21-producing CD4^+^ and CD8^+^ T cells in PB and SF of patients with RA and the lower panel summarizes the results (N=18). **(B)** Correlation of the percentage of IL-21^+^ CD4^+^ T cells with that of IL-21^+^ CD8^+^ T cells in RASF (N=18). **(C)** The panel summarizes the frequency of synovial IL-21-producing CD8^+^ T cells in RF-positive and -negative patients with RA. **(D)** The left panels are representative data of the percentage of CD45RA^-^PD-1^hi^ fraction among whole CD8^+^ T cells in OASF (N=3), RAPB (N=12) and RASF (N=18) and right panel summarizes the results. **(E)** The left panels are representative data of the percentage of IL-21-producing CD8^+^ T cells among CD45RA^+^, CD45RA^-^PD-1^-/low^, CD45RA^-^PD-1^int^ and CD45RA^-^PD-1^hi^ fractions in RASF and the right panel summarizes the results (N=12). *P < 0.05. **P < 0.01.

Based on the findings above, we next compared IL-21 production in CD4^+^ and CD8^+^ T cells from RAPB and RASF. As compared with HCPB (**Figure 1A**), there were significant numbers of IL-21-producing CD4^+^ and CD8^+^ T cells in RAPB in the absence of CD3/28 stimulation (**Figure 5A**). Notably, under these conditions, there were much more IL-21-producing CD4^+^ and CD8^+^ T cells in RASF than in RAPB (**Figure 5A**). In addition, the frequency of IL-21-producing CD8^+^ T cells significantly correlated with that of CD4^+^ T cells in RASF (**Figure 5B**). Previous studies showed that IL-21-producing CD4^+^ T (Tfh/Tph) cells are more frequently observed in seropositive RA patients (15, 31). Indeed, this was also the case in IL-21-producing CD8^+^ T cells: the ratio of IL-21-producing CD8^+^ T cells was higher in seropositive (RF^+^) RA patients than in seronegative (RF^-^) RA patients (**Figure 5C**). However, no correlation was found between the frequency of IL-21-producing CD8^+^ T cells and the number of swollen/tender joints, the titer of CRP, or anti-CCP (**Supplementary Figure 4A, B** **)**. Notably, CD45RA^-^PD-1^hi^CD8^+^ T cells were more abundant in RASF than in RAPB and SF from patients with osteoarthritis (OA) (**Figure 5D**). As similar to CD8^+^ T cells in HCPB (**Figure 2B**), IL-21 production was highest in CD45RA^-^PD-1^hi^CD8^+^ T cells in RASF (**Figure 5E**). These results suggest that in addition to CD4^+^ T cells, memory PD-1^hi^CD8^+^ T cells are a potent producer of IL-21 in RAPB and RASF.

### Memory PD-1^hi^CD8^+^ T cells Exhibit Similar Features to Tph Cells

**Figure 6:**
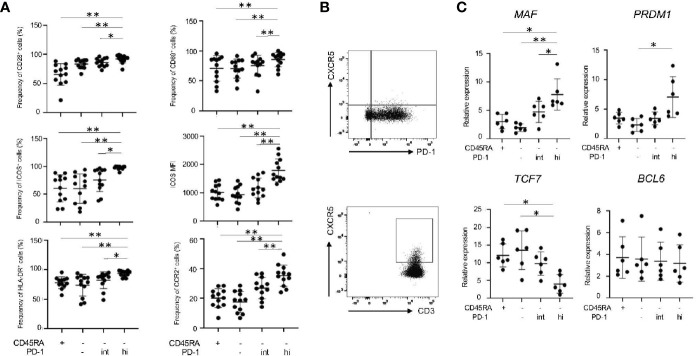
Memory PD-1^hi^ CD8^+^ T cells share several features with Tph cells in RASF. **(A)** The panels summarize the percentage of CD28^+^, CD69^+^, ICOS^+^, HLA-DR^+^ and CCR2^+^ among CD45RA^+^, CD45RA^-^PD-1^-/low^, CD45RA^-^PD-1^int^ and CD45RA^-^PD-1^hi^ fractions in RASF (N=12). **(B)** The upper panel shows the representative data regarding the percentage of CXCR5^-^CD8^+^ T cells in RASF and the lower panel shows representative data on CXCR5^+^CD4^+^ T cells in RAPB. **(C)** Comparison of levels of *MAF*, *PRDM1*, *TCF7*, *and BCL6* mRNA in CD45RA^+^, CD45RA^-^PD-1^-/low^, CD45RA^-^PD-1^int^ and CD45RA^-^PD-1^hi^ CD8^+^ T cells in RASF (N=6). *P < 0.05; **P < 0.01.

We further characterized IL-21-producing memory PD-1^hi^CD8^+^ T cells in RASF in terms of their expression of surface markers and transcriptional factors. PD-1^hi^CD8^+^ T cells in RASF expressed the highest levels of CD28^+^, CD69^+^, ICOS^+^ and HLA-DR^+^ (**Figure 6A**), a feature observed in IL-21-producing CD8^+^ T cells induced *in vitro* (**Figure 1C**). In addition, memory PD-1^hi^CD8^+^ T cells in RASF did not express CXCR5 (**Figure 6B**) but significantly expressed CCR2 (**Figure 6A**), whereas CD4^+^ T cells in RAPB expressed CXCR5 (**Figure 6B**), suggesting that PD-1^hi^CD8^+^ T cells in RASF share similar feature with Tph cells, but not Tfh cells, in terms of expression of chemokine receptors (5, 6, 15). Moreover, memory PD-1^hi^CD8^+^ T cells expressed the transcripts of *MAF* and *PRDM1* at high levels, while they expressed *TCF7* (encoding TCF-1) mRNA at low levels and *BCL6* mRNA at comparable levels among all CD8^+^ subsets (**Figure 6C**), the feature of which is similar to that of Tph cells (15, 16). These results suggest that memory PD-1^hi^CD8^+^ T cells in RASF exhibit similar features to Tph cells.

## Discussion

RA is a prototypical autoantibody-driven autoimmune disease in which T cell help to B cells is a fundamental event. In this respect, two distinct IL-21-producing CD4^+^ T cell subsets, Tfh and Tph cells, have recently gained much attention due to their potential for generating Ab-producing cells (5, 6, 15, 16). In this study, we showed that PD-1^hi^CD8^+^ T cells are also equipped to produce significant amounts of IL-21 that allows B cell differentiation, and these T cells were more abundant in RAPB and RASF as compared with HCPB.

A recent study showed that CD8^+^ T cells in RA synovium constitute PD-1^+^ and PD-1^-^ subsets wherein the latter only enriches granzyme-producing cytotoxic cells (20, 32). We found that PD-1^hi^CD8^+^ T cells were significantly enriched in the memory fraction that exhibit low levels of cytotoxic molecules in HC and RA patients (**Figures 2**, **5**). PD-1 is a coinhibitory molecule in activated T cells and a well-known marker of CD8^+^ T cell exhaustion, a condition that is characterized by impaired effector function and is frequently observed in the setting of both chronic viral infection and malignant tumors (33, 34). Recent evidence, however, suggests that PD-1^+^CD8^+^ T cells are not totally defective in their effector functions but rather exert unique functions. PD-1^+^CD8^+^ T cells are metabolically active and represent clonally expanding effectors in patients with juvenile idiopathic arthritis (35). Consistent with these findings, our current findings suggest that PD-1^+^CD8^+^ T cells actively contribute to RA pathogenesis *via* their production of IL-21.

One of our interests in this study was to elucidate the generation mechanism of IL-21-producing CD8^+^ T cells. Among the tested cytokines, combination of IL-12 and IL-21 most effectively generated IL-21-producing CD8^+^ T cells from naïve subsets *in vitro* (**Figure 3**). Notably, IL-21-producing CD8^+^ T cells co-produced IFN-γ but not IL-17 (**Figure 1B**). The IL-12-STAT4 pathway is the most potent in inducing both IFN-γ and IL-21 in human CD4^+^ T cells (36, 37). CD4^+^ T cells from patients deficient in IL-12 receptor β1 chain produced less IFN-γ and IL-21 upon stimulation (38). Given that Tfh1 and Tph cells produce large amounts of IFN-γ and IL-21 and they help B cells give rise to plasmablasts [6, 15], IL-21-producing CD8^+^ T cells may be equipped with similar features to Tfh1 and Tph cells. In addition to STAT4, STAT3-inducing cytokines such as IL-21 play a critical role in the generation of Tfh in humans (39). Together, these findings suggest that human CD8^+^ T cells share a similar mechanism of IL-21 production with CD4^+^ T cells. We found high levels of IL-21 production in CD8^+^ T cells in RASF (**Figure 5**). This may be explained by previous studies showing that *STAT4* SNP and STAT3 hyperactivation are associated with RA (40, 41), thus leading to accentuated response to IL-12 and IL-21.

Another compelling interest in this study was whether IL-21-producing memory PD-1^hi^CD8^+^ T cells in RA resemble the features of Tfh or Tph cells. Despite possession of B cell helper functions *via* IL-21 production, these cells exhibit distinct features. Tfh and Tph cells are characterized by the positivity of CXCR5 and CCR2, respectively. Compared with PB, RASF and RAST are significantly enriched with Tph cells (15, 16). We found that PD-1^hi^CD8^+^ T cells in RASF expressed CCR2, but not CXCR5 (**Figures 6A, B** **)**. Notably, this trend was also true for PD-1^hi^CD8^+^ T cells in RAPB (data not shown). BCL6 was originally reported as a pivotal TF in the generation of Tfh cells (42). Human cTfh cells, however, express BCL6 protein at very low levels, although a role of this TF in cTfh cells remains to be clarified. Instead, MAF is a critical TF in the function of Tfh and Tph cells, particularly for IL-21 production (15, 16, 43, 44). We found a clear correlation between MAF levels and IL-21 expression in PD-1^hi^CD8^+^ T cells of RASF (**Figure 6C**), again suggesting a conserved molecular mechanism of IL-21 induction between CD4^+^ and CD8^+^ T cells in humans. The expression of TCF-1 and BCL6 correlates inversely with that of BLIMP1 (45). Notably, Tph cells highly express BLIMP1, a transcription factor typically downregulated in Tfh cells (15, 17). We found that PD-1^hi^CD8^+^ T cells in RASF express BLIMP1 at high levels, while they express TCF1 at low levels, a trend observed in Tph cells (**Figure 6C**). Along with these characteristics, this suggests that memory PD-1^hi^CD8^+^ T cells in RASF are equipped with the features of Tph cells.

**Figure 7:**
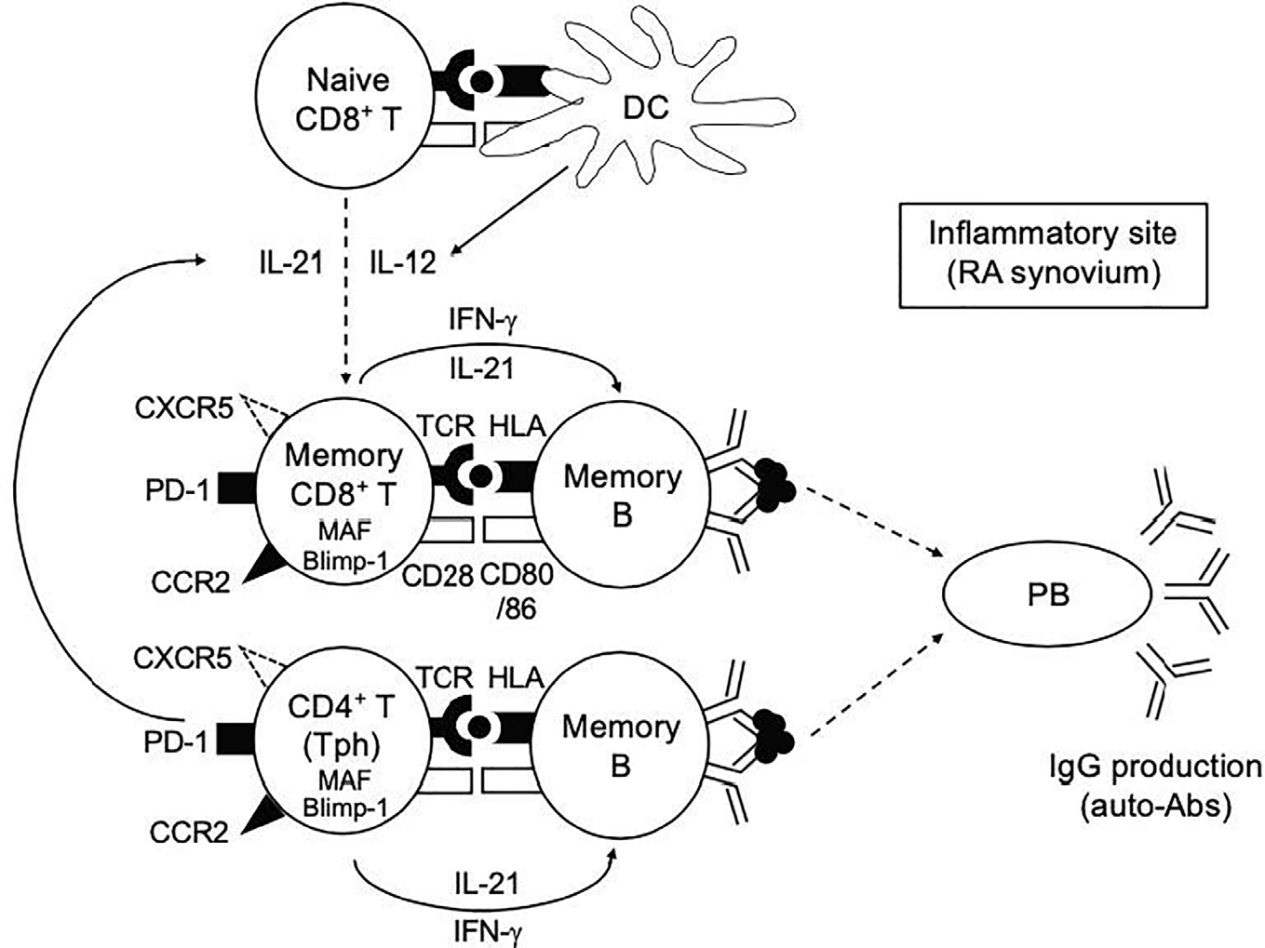
Our hypothetical model in this study. Both IL-12 and IL-21 play a pivotal role in the generation of IL-21-producing PD-1^+^CD8^+^ T cells. The provision of IL-12 and IL-21 to CD8^+^ T cells in this case could be from DCs and Tph cells, respectively. Memory PD-1^hi^CD8^+^ T cells, in concert with Tph cells, play a role in promoting plasmablast differentiation and antibody production at the inflammatory sites such as RA synovium.

What’s a role of PD-1^hi^CD8^+^ T cells in RA? IL-21 plays an essential role in the differentiation of B cells into Ab-secreting plasma cells (46). We showed that memory PD-1^hi^CD8^+^ T cells in HCPB upon CD3/28 stimulation promoted B cell differentiation into plasmablasts and IgG production in an IL-21-dependent manner, albeit to a bit lesser degree than memory PD-1^hi^CD4^+^ T cells (**Figure 4**). Based on these findings, we propose our hypothetical model depicted in **Figure 7**. Both IL-12 and IL-21 are critical cytokines that play a pivotal role in the generation of IL-21-producing PD-1^+^CD8^+^ T cells. Given the dominance of Tph over Tfh cells in RA synovium (15, 16), the provision of IL-21 to CD8^+^ T cells in this case could be from Tph cells. Indeed, the ratio of IL-21-producing CD8^+^ T cells correlated well with that of IL-21 producing CD4^+^ T cells in RASF (**Figure 5B**), implying the possibility that memory PD-1^hi^CD8^+^ T cells, in concert with Tph cells, promoted plasmablast differentiation in RASF, which is in accord with our finding that the ratio of IL-21-producing CD8^+^ T cells was significantly higher in seropositive (RF^+^) than seronegative (RF^-^) patients with RA (**Figure 5C**). Although correlation between the frequency of IL-21-producing CD8^+^ T cells and the number of swollen/tender joints, the titer of CRP and anti-CCP was not found (**Supplementary Figure 4A, B** **)**, an adequately powered clinical study is needed to confirm these findings.

The current study has several limitations. First, we still need to carefully elucidate whether memory PD-1^hi^CD8^+^ T cells in RASF are generated by the same mechanism as *in vitro* generated PD-1^hi^CD8^+^ T cells. IL-21 is shown to induce the expression of BCL6 and TCF-1 (47, 48), however mRNA levels of these genes in PD-1^hi^CD8^+^ T cells were not high in our results (**Figure 6C**), indicating the possibility that other cytokines asides from IL-12 and IL-21 are also involved in the generation of PD-1^hi^CD8^+^ T cells in RASF. Second, IL-21-producing CD8^+^ T cells are detected in the tissues of patients with nasal polyps and malignant tumors (22, 23). Intriguingly, however, these cells express high levels of CXCR5 which resemble the feature of Tfh. It thus remains to be determined whether Tfh-like CD8^+^ T cells also exist in patients with RA. Based on a recent study (20), this could be the case. Third, an intriguing issue of how much PD-1^hi^CD8^+^ T cells are involved in the pathogenesis of RA as compared with Tfh/Tph cells remains somewhat elusive at the moment. We showed the B-cell helper functions of PD-1^hi^CD8^+^ T cells in HCPB upon CD3/28 stimulation (**Figure 4**) as well as the existence and characteristics of PD-1^hi^CD8^+^ T cells in RASF (**Figures 5**, **6**). However, we still need more extensive analysis using more RASF samples to address this issue.

In conclusion, we demonstrate the characterization and generation mechanism of human IL-21-producing memory PD-1^hi^CD8^+^ T cells, which have similar features to Tph cells and accumulate abundantly in RASF. PD-1^+^CD8^+^ T cells characterized by impaired effector function are frequently observed in the setting of chronic infection and malignant tumors (33, 34), but our results suggest that memory PD-1^hi^CD8^+^ T cells in RASF, in concert with CD4^+^ T cells, play an active role in the pathogenesis of RA. Taken together, identification of this CD8^+^ T subset expands our knowledge of T cell subsets with B cell helper functions in RA, a prototypic systemic autoimmune disease. Selective targeting of these subsets could pave an avenue for the development of novel treatment strategies for this devastating disorder.

## Data Availability Statement

All data sets presented in this study are included in the article. Requests to access the datasets should be directed to niiro.hiroaki.811@m.kyushu-u.ac.jp.

## Ethics Statement

The Institutional Review Board of Kyushu University Hospital approved all research on human subjects (no 29-544). The patients/participants provided their written informed consent to participate in this study.

## Author Contributions

KH performed the experiments, statistical analysis, and drafted the manuscript. MY, MA, YK, HM, NO, YA, MK, TH, KA, and HN designed the study and helped to draft the manuscript. TS and HY provided synovial fluid cells of patients with RA and helped to draft the manuscript. HN contributed to data analysis and interpretation. All authors contributed to the article and approved the submitted version.

## Funding

This work was supported in part by Grants-in-Aid for Scientific Research from Japan Society for the Promotion of Science (HN: grant number 18K08410).

## Conflict of Interest

The authors declare that the research was conducted in the absence of any commercial or financial relationships that could be construed as a potential conflict of interest.
