# Supplementary material for: Human PD-1hiCD8+ T Cells Are a Cellular Source of IL-21 in Rheumatoid Arthritis
Source: Front Immunol. 2021 Mar 19;12:654623. doi: 10.3389/fimmu.2021.654623 (PMC8017303; doi:10.3389/fimmu.2021.654623)
Supplement: Supplementary file 1 [file DataSheet_1.docx]

Supplementary Material

## Supplementary Figures and Table


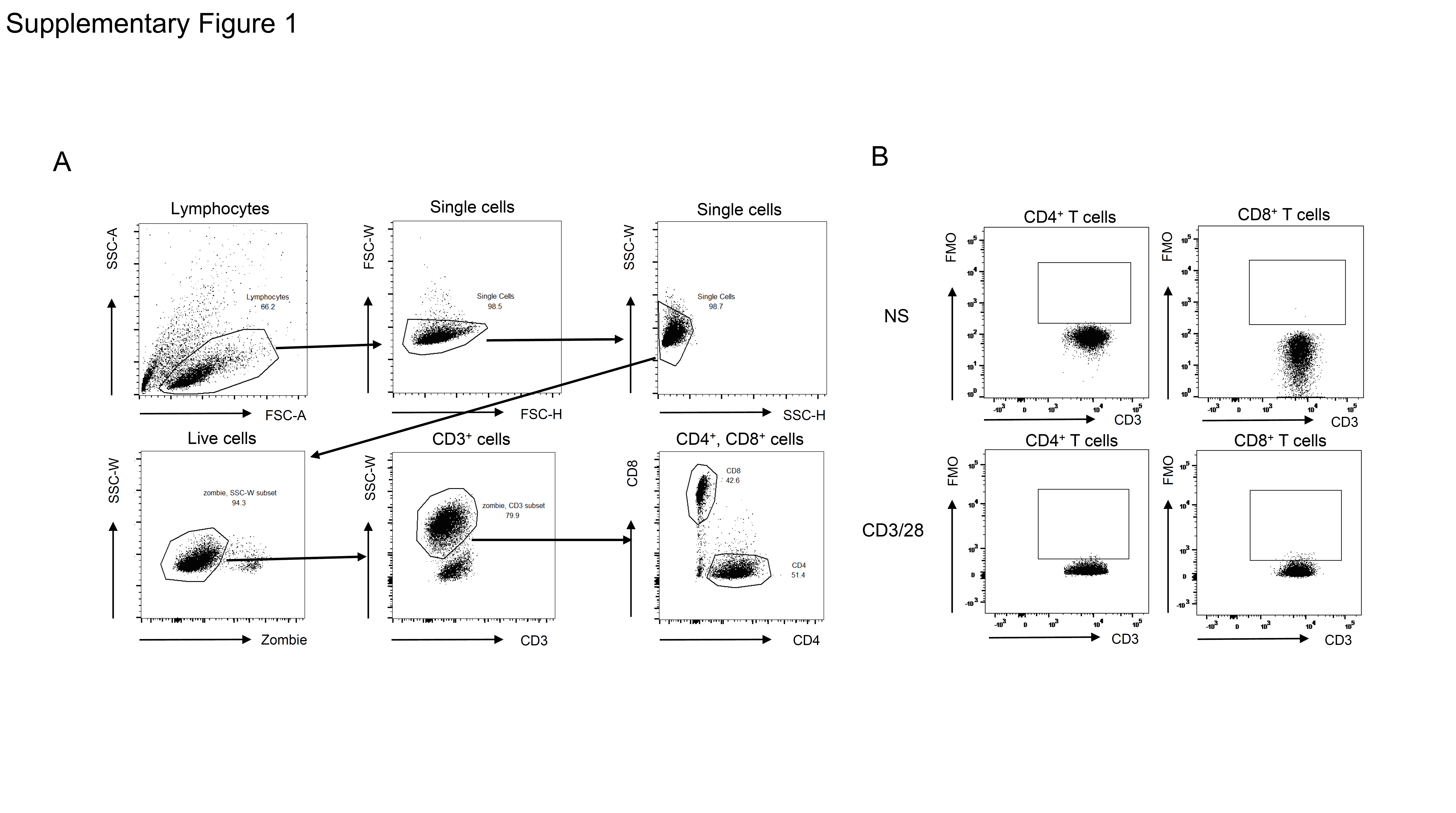


**SUPPLEMENTARY FIGURE 1** The representative gating strategy and FMO control. **(A)** The representative overview of gating strategy in Figure 1 is shown.  **(B)** The representative FMO control panels of IL-21 in CD4^+^ and CD8^+^ T cells with or without CD3/28 stimulation are shown.


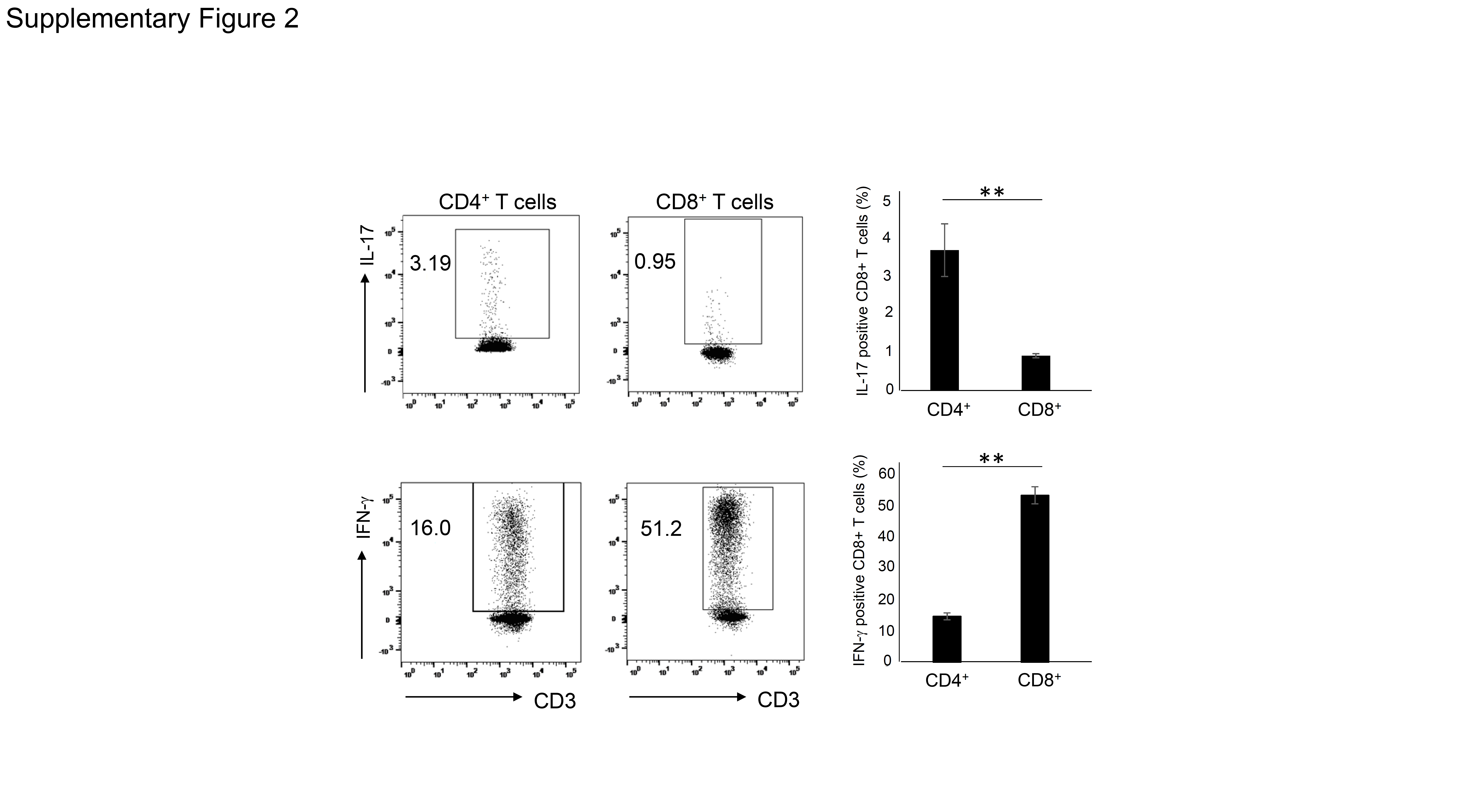


**SUPPLEMENTARY FIGURE 2** The production of IFN-γ and IL-17 in CD4^+^ and CD8^+^ T cells. Left panels show the representative data of IFN-γ and IL-17-producing CD4^+^ and CD8^+^ T cells in HCPB upon CD3/28 stimulation. The right graphs summarize the results (N=4). ***P* < 0.01.


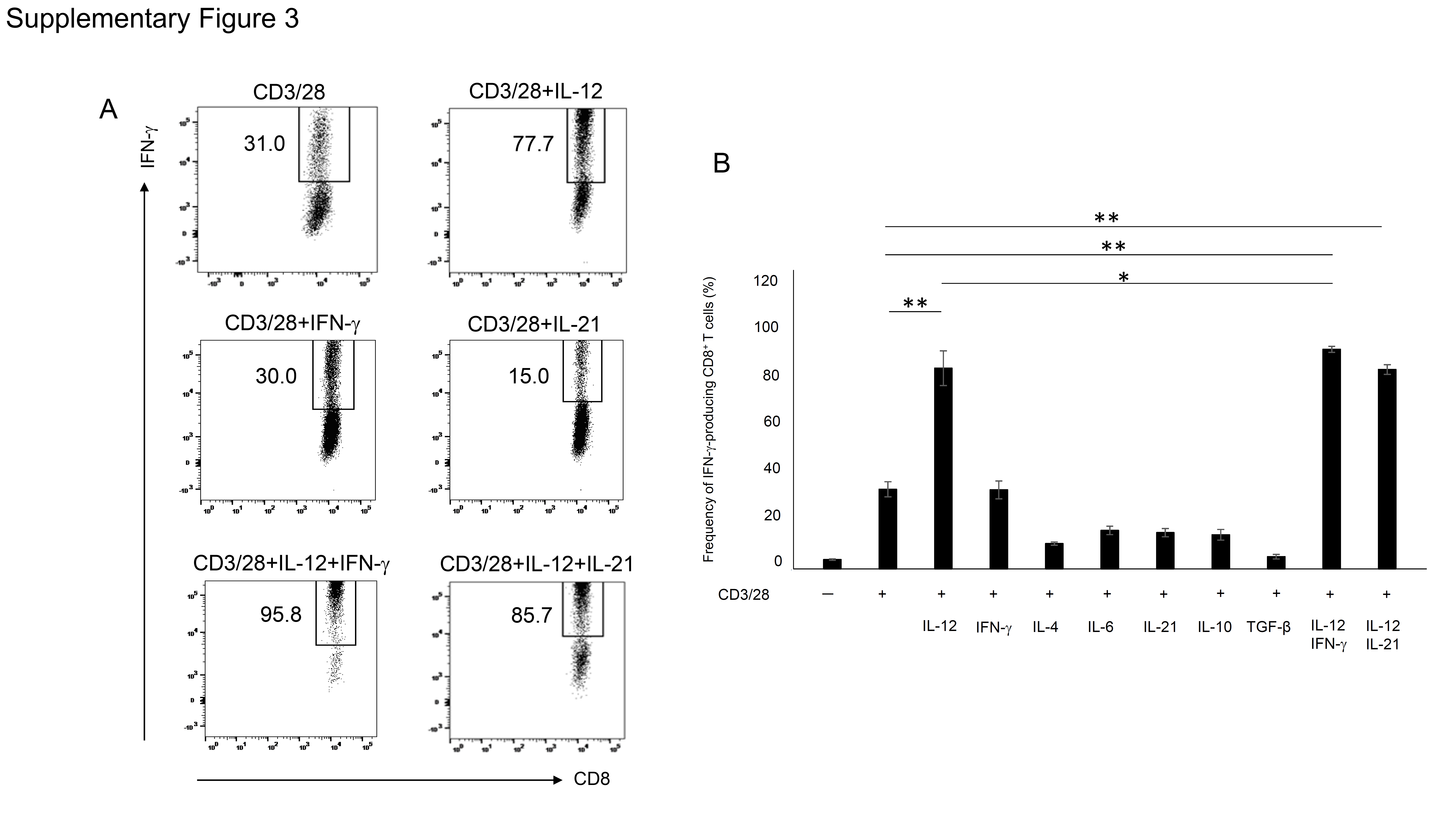


**SUPPLEMENTARY FIGURE 3** IL-12 induces IFN-γ-producing T cells from naïve CD8^+^ T cells in HCPB. **(A)** The panels show the representative data regarding the percentage of IFN-γ-producing CD8^+^ T cells in HCPB under indicated conditions. (**B)** The graph summarizes the results of **A** (N=4). **P* < 0.05; ***P* < 0.01.


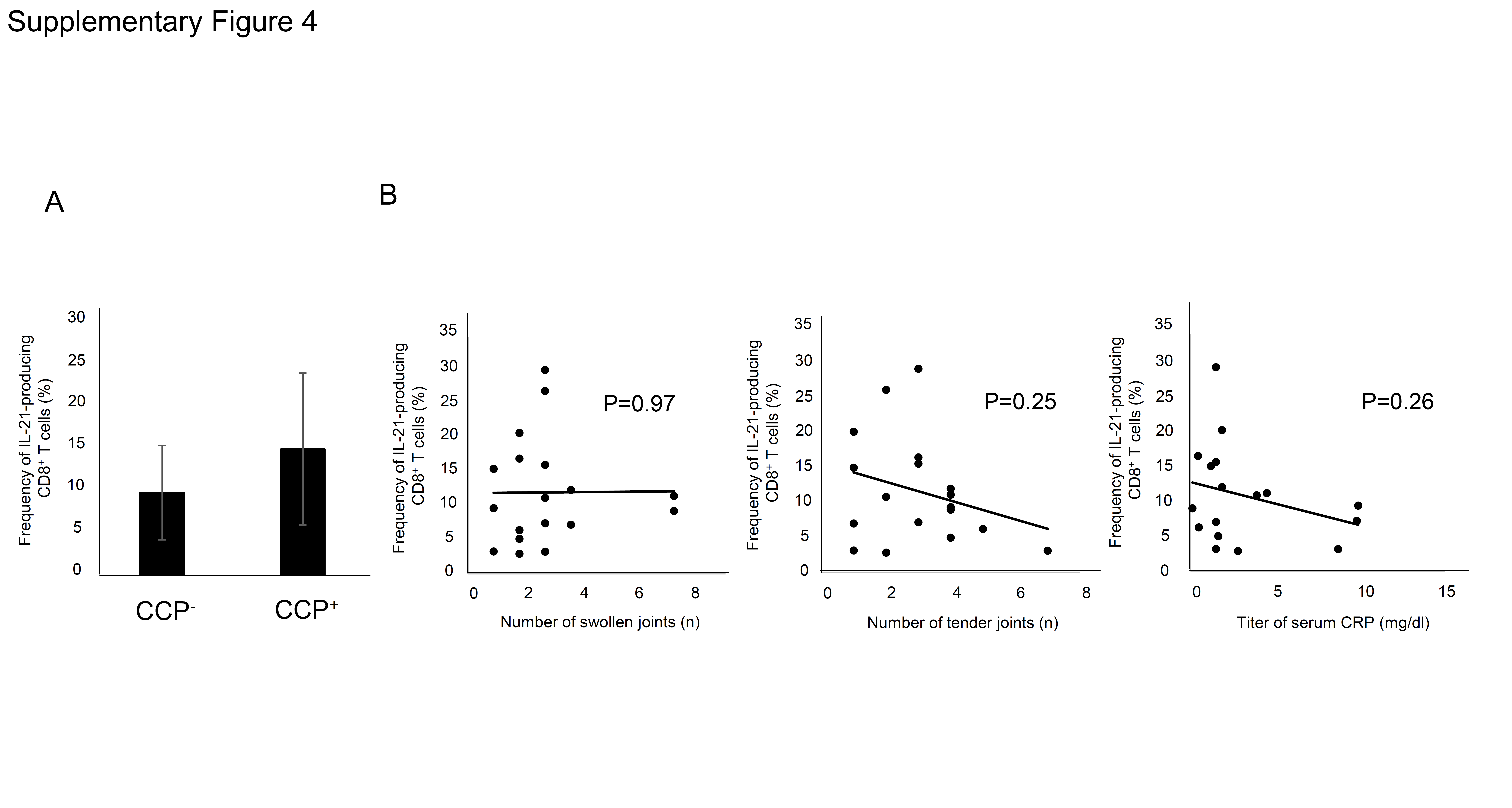


**SUPPLEMENTARY FIGURE 4** The relationship between the frequency of synovial IL-21-producing CD8^+^ T cells and the clinical data. **(A)** The graph summarizes the ratio of IL-21-producing CD8^+^ T cells in RASF in anti-CCP^-^ and anti-CCP^+^ patients with RA. (**B)** The panels show the correlation between the ratio of IL-21-producing CD8^+^ T cells in RASF and number of swollen/tender joints and titer of serum CRP in patients with RA.

**SUPPLEMENTARY TABLE 1** Baseline characteristics.
